# One-Step Assembly of a PRRSV Infectious cDNA Clone and a Convenient CRISPR/Cas9-Based Gene-Editing Technology for Manipulation of PRRSV Genome

**DOI:** 10.3390/v15091816

**Published:** 2023-08-26

**Authors:** Hejin Zhang, Kaiqi Duan, Yingbin Du, Shaobo Xiao, Liurong Fang, Yanrong Zhou

**Affiliations:** 1 State Key Laboratory of Agricultural Microbiology, College of Veterinary Medicine, Huazhong Agricultural University, Wuhan 430070, China; zhj1993@webmail.hzau.edu.cn (H.Z.); duankq@webmail.hzau.edu.cn (K.D.); dyblky@webmail.hzau.edu.cn (Y.D.); vet@mail.hzau.edu.cn (S.X.); fanglr@mail.hzau.edu.cn (L.F.); 2 The Key Laboratory of Preventive Veterinary Medicine in Hubei Province, Cooperative Innovation Center for Sustainable Pig Production, Wuhan 430070, China

**Keywords:** PRRSV, reverse genetics system, CRISPR/Cas9, gene-editing

## Abstract

Porcine reproductive and respiratory syndrome (PRRS) has been a persistent challenge for the swine industry for over three decades due to the lack of effective treatments and vaccines. Reverse genetics systems have been extensively employed to build rapid drug screening platforms and develop genetically engineered vaccines. Herein, we rescued recombinant PRRS virus (rPRRSV) WUH3 using an infectious cDNA clone of PRRSV WUH3 acquired through a BstXI-based one-step-assembly approach. The rPRRSV WUH3 and its parental PRRSV WUH3 share similar plaque sizes and multiple-step growth curves. Previously, gene-editing of viral genomes depends on appropriate restrictive endonucleases, which are arduous to select in some specific viral genes. Thus, we developed a restrictive endonucleases-free method based on CRISPR/Cas9 to edit the PRRSV genome. Using this method, we successfully inserted the exogenous gene (EGFP gene as an example) into the interval between ORF1b and ORF2a of the PRRSV genome to generate rPRRSV WUH3-EGFP, or precisely mutated the lysine (K) at position 150 of PRRSV nsp1α to glutamine (Q) to acquire rPRRSV WUH3 nsp1α-K150Q. Taken together, our study provides a rapid and convenient method for the development of genetically engineered vaccines against PRRSV and the study on the functions of PRRSV genes.

## 1. Introduction

Porcine reproductive and respiratory syndrome virus (PRRSV), which first emerged in the United States in the late 1980s, has now spread worldwide [1]. PRRSV is an enveloped RNA virus belonging to the family *Arteriviridae* of the order *Nidovirales* [2]. According to the viral diversity of its antigenicity and genomes, PRRSV is classified into two genotypes, European (EU)-type and North American (NA)-type, which only share approximately 65% sequence identity in genome [3,4]. The PRRSV genome is a single-stranded, positive-sense RNA of ~15 kb in length, comprising a 5’ cap structure, a 3’ poly (A) tail and 10 open reading frames (ORFs). The ORF1a and ORF1b occupy the 5’-proximal 75% of the genome and encode two polyproteins (pp1a and pp1ab), which are ultimately cleaved into at least 16 nonstructural proteins (nsps) [5,6,7]. The remaining 3’-proximal 25% of the PRRSV genome encodes viral structural proteins, including glycoprotein 2 (GP2), envelope protein (E), GP3, GP4, GP5, ORF5a protein, membrane protein (M), and nucleocapsid protein (N) [8,9,10,11,12].

PRRSV primarily causes abortion in pregnant sows and respiratory distress in pigs of all ages, resulting in a tremendous burden on the global swine industry [13,14]. But the effective vaccine and specific drug against PRRSV are still unavailable currently; thus, it is essential to genetically lucubrate the viral pathogenesis and develop a new generation of enhanced vaccines. The reverse genetics system is considered an efficient tool for the function studies of viral genes [15,16]. By knocking out the E gene in the PRRSV genome, it demonstrates that E protein is involved in the uncoating process of virus infection rather than in the assembly process [17]. Through a deletion of a stretch of 34 nucleotides (14,653 to 14,686) within ORF7 using the viral reverse genetics system, this fragment of ORF7 has been demonstrated to be critical for the synthesis of PRRSV negative-strand genomic RNA [18]. In addition, the reverse genetics system is also applied to the development of genetically engineered vaccines. Presently, many live-attenuated vaccine candidates are generated by altering the virulence genes in the PRRSV genome through the reverse genetics systems [19]. Additionally, an immunogenically enhancing vaccine candidate that possesses a stronger ability to induce neutralizing antibody has been developed by mutating the glycosylation sites in GP5 [20,21,22]. Moreover, four regions in the PRRSV genome, including the interval between ORF1b and ORF2, the spacer between ORF4 and ORF5a, the position post-ORF7, and the highly variable region within nsp2, have been applied for carrying exogenous genes using a reverse genetics system, which provides the potential for the development of polyvalent vaccines based on PRRSV [23,24,25,26].

Traditional reverse genetics systems rely on specific restrictive endonuclease or homologous recombination, the time-consuming disadvantage and the lack of appropriate restrictive endonucleases in certain genes limit the application of a reverse genetics system. Therefore, it is necessary to improve this method. CRISPR/Cas9, a novel gene-editing technology derived from bacteria and archaea [27], significantly increases the selection freedom of the gene modification position [23,26] and is more time-saving [28]. CRISPR/Cas9 has been widely used to edit the genomes of various species [29,30,31]. In terms of viral genome editing, CRISPR/Cas9 is successfully used to edit the genomes of DNA viruses, especially some carcinogenic viruses and herpesviruses, such as hepatitis B virus, human papillomavirus, herpes simplex virus 1, and pseudorabies virus (PRV) [32,33,34]. For example, a gE/gI/TK three-gene inactivated PRV HeN1 strain was successfully constructed using CRISPR/Cas9, which provides enhanced protection for susceptible animals compared to the traditional PRV strain Bartha-K61 [35]. Moreover, a trivalent vaccine strain against duck enteritis virus (DEV), duck Tembusu virus, and the highly pathogenic avian influenza virus H5N1 was also developed using CRISPR/Cas9 based on DEV [36]. However, CRISPR/Cas9 has rarely been utilized for the editing of RNA virus genomes. To date, only the insertion of the EGFP gene into the porcine epidemic diarrhea virus genome and the deletion of the spike gene *N*-terminal domain from the transmissible gastroenteritis virus genome via CRISPR/Cas9 have been reported [37,38].

Presently, there is no report on the application of CRISPR/Cas9 in PRRSV genome editing. In this research, we first constructed a full-length infectious cDNA clone of the PRRSV strain WUH3 using a one-step-assembly approach based on the restriction enzyme BstXI and successfully rescued the recombinant virus rPRRSV WUH3. Subsequently, the CRISPR/Cas9 technology was used to edit the PRRSV genome. By inserting the EGFP gene into the PRRSV genome or mutating lysine (K) at the position of 150 of PRRSV nsp1α to glutamine (Q), we acquired two recombinant viruses, rPRRSV WUH3-EGFP and rPRRSV WUH3 nsp1α-K150Q. In conclusion, this study provides an efficient, convenient, and CRISPR/Cas9-based gene-editing technique for the manipulation of the PRRSV genome.

## 2. Materials and Methods

### 2.1. Cells and Viruses

Monkey kidney (MARC-145) cells were cultured in Dulbecco’s Modified Eagle’s Medium (DMEM; Invitrogen, Carlsbad, CA, USA) supplemented with 10% fetal bovine serum (FBS; Gibco, Grand Island, NY, USA) in a 37 °C, 5% CO_2_ humidified incubator. A highly pathogenic PRRSV (HP-PRRSV) strain WUH3 (GenBank Accession No. HM853673) was isolated from an ill pig with “high fever” in China [39].

### 2.2. Selection of Restrictive Endonuclease in Genome and Assembly of a Full-Length cDNA Clone

The restrictive endonuclease enzyme, BstXI (R0113V; NEB, MA, USA) recognizing 5′···CCANNNNNNTGG···3′, was selected for constructing full-length infectious clones after analyzing genomes of HP-PRRSV WUH3 strain. A plaque purified PRRSV WUH3 was used as the parent virus, and six viral gene fragments (F1: nt 1–372; F2: nt 352–1541; F3: nt 1521–4806; F4: nt 4786–7548; F5: nt 7530–13,778; F6: nt 13,528–15,330) were amplified by RT-PCR using HiScript II 1st Strand cDNA Synthesis Kit and 2 × Phanta Flash Master Mix (Vazyme, Nanjing, China). Meanwhile, some regulatory elements including HHR (hammerhead ribozyme), HDVrz (Hepatitis Delta Virus ribozyme), and BGH (bovine growth hormone) transcript termination signal were incorporated into pCMV vector. To assemble successfully, F1 and F6 fragments were fused with the above-modified pCMV vector (F6-pCMV-F1). Then, F6-pCMV-F1 as well as F2 to F5 were ligated into a topological cloning vector pCE-Blunt (Vazyme) for sequencing. Purified viral gene fragments digested from the pCE-Blunt vector were ligated by T4 ligase (Vazyme) for assembling the PRRSV WUH3 full-length cDNA clone. The assembled products were transformed into competent Escherichia coli cells DH10B. After 16 h culture, monoclonal transformants were picked out and cultured in liquid medium supplemented with 50 μg/mL of Kanamycin (Beyotime, Shanghai, China) to extract plasmids using Plasmid Extract Kits (Omega, Norcross, GA, USA), and the molecular weight sizes of plasmids were tested using DNA gel electrophoresis. The plasmid with the molecular weight of ~19 kb was further verified using restriction fragment length polymorphism (RFLP) using BstXI.

### 2.3. Recovery of Recombinant PRRSV

MARC-145 cells were seeded in six-well plates and cultured in a 37 °C, 5% CO_2_ humidified incubator. The correct pCMV-WUH3 plasmid was transfected into cells using Lipofectamine 3000 (Invitrogen). Six hours after transfection, the cells were washed three times with DMEM and supplemented with 2 mL of DMEM containing 2% FBS. Finally, the cells were cultured in a 37 °C, 5% CO_2_ humidified incubator for 4–5 days until typical cytopathic effect (CPE) was observed.

### 2.4. Design and Synthesis of sgRNAs

The design of sgRNAs for cleaving pCMV-WUH3 was performed using the online tool provided by Integrated DNA Technologies on 2 March 2022 (https://sg.idtdna.com/site/order/designtool/index/CRISPR_PREDESIGN). Briefly, nucleotide sequences within approximately 100 base pairs (bp) surrounding the gene-editing site were inputted in FASTA format into the tool. The sgRNA with the highest on-target score was selected to induce cleavage in pCMV-WUH3. The sgRNA templates were synthesized using 2 × Taq Master Mix (Vazyme) along with scaffold oligo and specific primers (ssDNAa-ORF1b or ssDNAb-ORF2a, and ssDNAa-nsp1α or ssDNAb-nsp1α). Transcription in vitro was then performed using a T7 transcription kit (NEB) at 37 °C.

### 2.5. Analysis of Residue Conservation in PRRSV WUH3 nsp1α

To identify the appropriate residue for mutation at PRRSV WUH3 nsp1α-K150, we randomly downloaded 42 nsp1α sequences of different PRRSV-EU and PRRSV-NA strains from the NCBI nucleotide database on 17 June 2022 (https://www.ncbi.nlm.nih.gov/nuccore). To assess the genetic evolutionary relationships among nsp1α in different genotypes of PRRSV, we performed phylogenetic analysis using MEGA 7.05 software. Furthermore, we utilized the online tool ESPript to visualize the conservation of the residue at position 150 of nsp1α across various PRRSV isolates on 17 June 2022 (https://espript.ibcp.fr/ESPript/ESPript/index.php).

### 2.6. Construction of pCMV-WUH3-EGFP and pCMV-WUH3 nsp1α-K150Q

The pCMV-WUH3 plasmids were cleaved in the mixture comprising 3 μg of pCMV-WUH3, 2 μL of Cas9 nuclease (Vazyme), 10 μL of sgRNAs product (5 μL for each sgRNA), 5 μL of 10× Cas9 nuclease reaction buffer, and RNase-free water up to a final volume of 50 μL. The reactions were incubated at 37 °C for 3 h. Subsequently, the incised pCMV-WUH3 was purified by isopropanol precipitation and verified through electrophoresis in 1% agarose gel. For the construction of the recombinant plasmid pCMV-WUH3-EGFP, a ClonExpress Ultra One-Step Cloning Kit (Vazyme) was employed. The construction process involved the mixing of the cleaved pCMV-WUH3 and an EGFP gene flanked by partial PRRSV WUH3 ORF1b and ORF2a. Similarly, the recombinant plasmid pCMV-WUH3 nsp1α-K150Q was constructed by recombining the cleaved pCMV-WUH3 with a mutant nsp1α-K150Q gene.

### 2.7. Indirect Immunofluorescence Assay (IFA)

MARC-145 cells were infected with PRRSV at a multiplicity of infection (MOI) of 0.1 or transfected with an infectious cDNA clone. After 24 h, the cells were fixed with 4% paraformaldehyde for 15 min, then permeabilized with methanol for 10 min. Next, the cells were washed three times with phosphate-buffered saline (PBS) and blocked using 5% bovine serum albumin (BSA) at 37 °C for 1 h. Following three washes with PBS, the cells were incubated with monoclonal antibody specific to PRRSV-N protein at 37 °C for 1 h. Afterwards, the cells were stained with Alexa Fluor 488-labeled Goat Anti-Mouse IgG (Beyotime) at 37 °C for 45 min. The nuclei were stained with 0.01% 4′,6-diamidino-2-phenylindole for 15 min. After three washes with PBS, fluorescent images were visualized and collected using a fluorescence microscope (Nikon, Tokyo, Japan).

### 2.8. Western Blotting Assay

MARC-145 cells seeded in six-well plates were infected with PRRSV. At 24 hpi, the cells were harvested with 1 × SDS loading buffer (Beyotime) and boiled for 10 min. After centrifuging at 12,000× *g*, 4 °C for 10 min, proteins were separated by electrophoresis on 12% polyacrylamide gels (EpiZyme Biotechnology, Shanghai, China) and electro-blotted onto polyvinylidene difluoride membranes (Millipore, MA, USA). Next, the membranes were blocked at room temperature with 5% BSA for 3 h and incubated with the antibody specific to PRRSV-N protein, followed by incubation with horseradish peroxidase-conjugated Goat Anti-Mouse IgG (Beyotime). Finally, the resultant images were visualized and collected using Clarity Western Peroxide Reagent (Bio-Rad, CA, USA).

### 2.9. Viral Plaque Assay

MARC-145 cells seeded in six-well plates were incubated with 800 μL of the serial 10-fold dilutions of PRRSV samples for 2 h at 4 °C, then washed three times with PBS. The plaque-forming liquid, which was prepared at 42 °C by mixing 1.8% low-melting-point agarose (Beyotime) and 2× phenol-red free DMEM (Invitrogen) in equal volume, was added to the six-well plates. Next, the plates were placed at 4 °C for 15 min. Finally, the cells were cultured in an incubator at 37 °C. When the viral plaques were observed, 1% crystal violet solution (Beyotime) was added to the six-well plates to stain viral plaques at 37 °C for 3 h, then the low-melting-point agarose was gently washed away with running water.

### 2.10. Viral Multiple-Step Growth Curves Measured by TCID_50_ Assay

MARC-145 cells in 24-well plates were infected with PRRSV (0.1 MOI). After 1 h of incubation at 37 °C for virus attachment, the supernatant was discarded. Cells were washed three times with PBS, and 1 mL of DMEM containing 2% FBS was added to each well. Samples were collected at indicated time points after infection for the detection of virus titers using TCID_50_ assays. The titers of PRRSV were calculated using the Reed–Muench method and shown as the TCID_50_/mL. Viral multiple-step growth curve was visualized using GraphPad Prism 7.05 software (GraphPad Software, San Diego, CA, USA).

### 2.11. Statistical Analysis

GraphPad Prism 7.05 software (GraphPad Software) was used for data analysis using Students’ *T* test or one-way ANOVA.

## 3. Results

### 3.1. Assembly of Full-Length Infectious cDNA Clone of PRRSV WUH3

**Figure 1:**
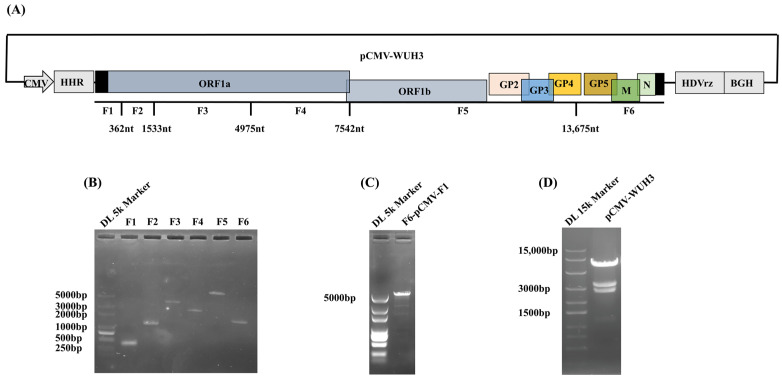
Assembly of full-length infectious cDNA clone of PRRSV WUH3. (**A**) The PRRSV WUH3 genome was divided into six contiguous gene fragments (F1 to F6) based on the distribution of BstX I site. (**B**) Amplification of the six fragments of PRRSV WUH3. The gene fragments of PRRSV WUH3 were amplified using RT-PCR with specific primers. (**C**) Fusion of F1, the modified pCMV, and F6 to construct F6-pCMV-F1. (**D**) RFLP analysis of pCMV-WUH3 using BstXI.

The full-length genome of PRRSV WUH3 was divided into six segments, labeled as F1 to F6, based on the BstXI sites (Figure 1A). Firstly, to transcribe the viral genome correctly, specific transcript regulating elements, including the HHR, HDVrz, and BGH transcript termination signal, were introduced into the pCMV vector. Meanwhile, six viral fragments (F1 to F6) were successfully cloned (Figure 1B) with specific primers as shown in Table 1. Subsequently, the F1 and F6 fragments were fused with the above-modified pCMV vector to form F6-pCMV-F1 (Figure 1C). The fragments (F2 to F5) and F6-pCMV-F1 were ligated into the cloning vector pCE-Blunt and determined using Sanger sequencing. These fragments were then excised from pCE-Blunt and assembled using T4 ligase to construct the full-length infectious cDNA clone of PRRSV WUH3 (pCMV-WUH3). Finally, the pCMV-WUH3 was verified using RFLP with BstXI (Figure 1D).

### 3.2. Recovery, Identification, and Characterization of rPRRSV WUH3

**Figure 2:**
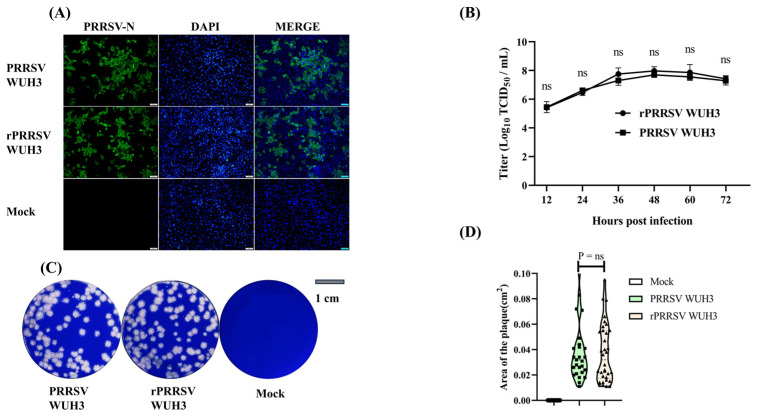
Recovery, identification, and characterization of rPRRSV WUH3. (**A**) Expression of PRRSV-N protein in MARC-145 cells transfected with pCMV-WUH3 was detected by IFA at 5 days post-transfection. Additionally, cells were infected with PRRSV WUH3 (0.1 MOI), and PRRSV-N protein expression was assessed at 24 hpi. (**B**) The multiple-step growth curves of PRRSV WUH3 and rPRRSV WUH3 at an MOI of 0.1 were analyzed using TCID_50_ assays. (**C**) Plaques formed by PRRSV WUH3 and rPRRSV WUH3 in MARC-145 cells were stained with 1% crystal violet at 96 hpi. (**D**) Areas of plaques formed by PRRSV WUH3 and rPRRSV WUH3 were detected using Image J and compared using GraphPad Prism 7. Data are displayed in a Violin Plot. ns, *p* > 0.5.

To rescue the recombinant PRRSV (rPRRSV) WUH3, the pCMV-WUH3 plasmid was transfected into MARC-145 cells using Lipofectamine 3000. After 4–6 days post-transfection, PRRSV-induced CPE characterized by cellular rounding and clumping was observed. Furthermore, the expression of the PRRSV-N protein was confirmed using IFA (Figure 2A). To assess whether rPRRSV WUH3 exhibited similar growth kinetics to its parental virus (PRRSV WUH3) in vitro, viral proliferation was evaluated with multiple-step growth assays. MARC-145 cells were infected with rPRRSV WUH3 or PRRSV WUH3 at an MOI of 0.1. The virus titers were determined using TCID_50_ assays at 12, 24, 36, 48, 60, and 72 h post-infection (hpi). The results showed that the titers of rPRRSV WUH3 were slightly higher than those of PRRSV WUH3 after 24 h, but the titers of both strains reached their highest at 48 hpi (Figure 2B). Additionally, rPRRSV WUH3 formed plaques similar in size to those of PRRSV WUH3 (Figure 2C,D).

### 3.3. Modification of PRRSV Genome Using CRISPR/Cas9 to Carry an Exogenous Gene

**Figure 3:**
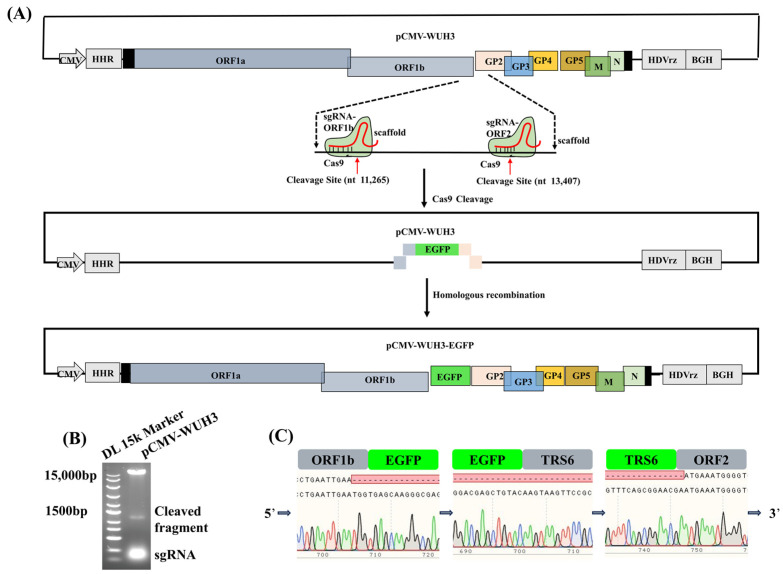
Insertion of the EGFP gene into PRRSV genome using CRISPR/Cas9. (**A**) Schematic diagram of modification of PRRSV genome by cleaving pCMV-WUH3 with CRISPR/Cas9 and subsequently ligating the EGFP gene into the cleaved pCMV-WUH3 through homologous recombination. (**B**) The cleavage of pCMV-WUH3 by CRISPR/Cas9 was examined using electrophoresis in 1% agarose gel. (**C**) The constructed pCMV-WUH3-EGFP was verified using Sanger sequencing.

Recombinant viruses containing EGFP are convenient tools for the assessment of viral proliferation. We, thus, here, used the EGFP gene as a model to develop a platform for rapid modification of the PRRSV genome. The EGFP gene was scheduled to be inserted into the interval between ORF1b and ORF2 of PRRSV WUH3 using CRISPR/Cas9 (Figure 3A). To linearize pCMV-WUH3, sgRNAs targeting ORF1b or ORF2, were designed using the online tool on 2 March 2022 (https://sg.idtdna.com/site/order/designtool/index/CRISPR_PREDESIGN) and synthesized using oligos as given in Table 2. Two sgRNAs targeting ORF1b and ORF2 with the highest on-target scores, named sgRNA-ORF1b and sgRNA-ORF2, respectively, were selected to cleave the pCMV-WUH3 plasmid. The result showed that the Cas9/sgRNAs complex specifically cleaved the pCMV-WUH3 into DNA fragments of about 1.2 kb and linearized pCMV-WUH3 (Figure 3B). Then, the EGFP gene was ligated to the linearized pCMV-WUH3 by homologous recombination to form pCMV-WUH3-EGFP, which was further confirmed using Sanger sequencing (Figure 3C).

### 3.4. Rescue and Characteristics of rPRRSV WUH3-EGFP

**Figure 4:**
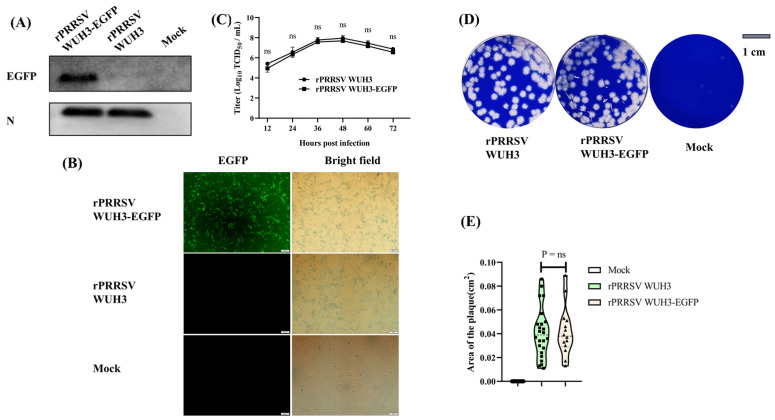
Rescue and characteristics of rPRRSV WUH3-EGFP. (**A**) The expression of EGFP and PRRSV-N protein in MARC-145 cells transfected with pCMV-WUH3-EGFP or pCMV-WUH3 was examined using Western blotting assays. (**B**) MARC-145 cells were transfected with pCMV-WUH3-EGFP or pCMV-WUH3 for 5 days, the typical PRRSV CPE in MARC-145 cells and the green fluorescence were observed using a fluorescence microscope. (**C**) The multiple-step growth curves of rPRRSV WUH3-EGFP and rPRRSV WUH3 in MARC-145 at an MOI of 0.1 were analyzed using TCID_50_ assays. (**D**) Plaques formed by rPRRSV WUH3-EGFP and rPRRSV WUH3 in MARC-145 cells were stained with 1% crystal violet at 96 hpi. (**E**) Areas of plaques formed by rPRRSV WUH3-EGFP and rPRRSV WUH3 were detected using Image J and compared using GraphPad Prism 7, respectively. Data are displayed in a Violin Plot. ns, *p* > 0.5.

The pCMV-WUH3-EGFP or pCMV-WUH3 was transfected into MARC-145 cells to generate rPRRSV WUH3-EGFP or rPRRSV WUH3, respectively. After 5 days, typical PRRSV CPE was observed in cells transfected with both pCMV-WUH3-EGFP and pCMV-WUH3. The expression of EGFP and PRRSV-N protein was confirmed using Western blotting assays. The results showed that PRRSV-N protein was expressed in cells transfected with both pCMV-WUH3-EGFP and pCMV-WUH3, while the EGFP expression was only detected in cells transfected with pCMV-WUH3-EGFP, rather than the cells transfected with pCMV-WUH3 (Figure 4A). Furthermore, green fluorescence emitted by EGFP could be observed in cells transfected with pCMV-WUH3-EGFP, but not in cells transfected with pCMV-WUH3 (Figure 4B). The growth kinetics of rPRRSV WUH3-EGFP and rPRRSV WUH3 in MARC-145 cells were examined through TCID_50_ assays, and the results showed that the proliferation of both rPRRSVs peaked at 48 hpi. However, a slightly lower titer was observed for rPRRSV WUH3-EGFP at all tested time points compared to rPRRSV WUH3 (Figure 4C). Additionally, the rPRRSV WUH3-EGFP virus exhibited comparable plaque size to that of rPRRSV WUH3 in MARC-145 cells (Figure 4D,E). These results indicate that CRISPR/Cas9 could efficiently insert exogenous genes into PRRSV genomes.

### 3.5. Site-Directed Mutation of PRRSV Genome Using CRISPR/Cas9

To provide support for the function study of the potentially critical amino acids in specific viral genes, we attempted to further present a protocol for site-directed mutation of the PRRSV genome. Although CRISPR/Cas9 has been used to generate precise mutations in the genomes of various species, its application in viral genomes has not been reported. Herein, we tried to construct site-mutated rPRRSV using CRISPR/Cas9 technology.

**Figure 5:**
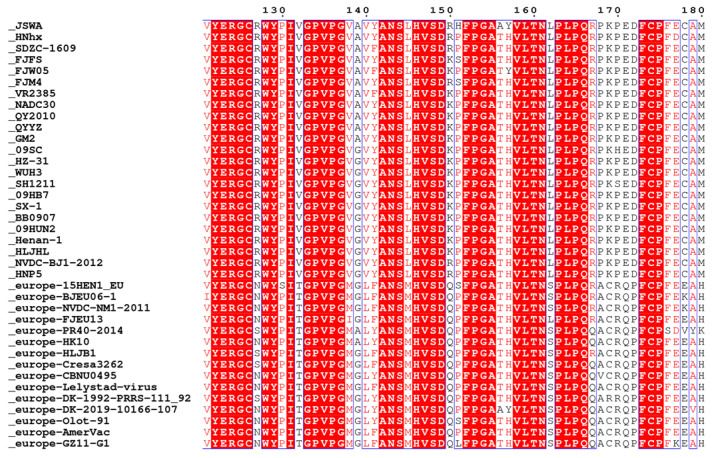
The residue conservation analysis at the position of 150 in nsp1α of PRRSV isolates.

It has been reported that ubiquitination of PRRSV nsp1α is associated with its immunosuppressive function. Three potential ubiquitination modification sites exist in the nsp1α of PRRSV WUH3, including lysine (K) residues at position of 117, 150, and 169. Since the function of K117 and K169 has been studied [40,41], we focused on K150. Firstly, the conservation of K150 in nsp1α between NA and EU PRRSV strains was analyzed. We observed that K150 in nsp1α is mostly K in NA-type PRRSV and Q in EU-type PRRSV (Figure 5). Thus, we planned to mutate K150 to Q150 in the nsp1α of PRRSV WUH3.

**Figure 6:**
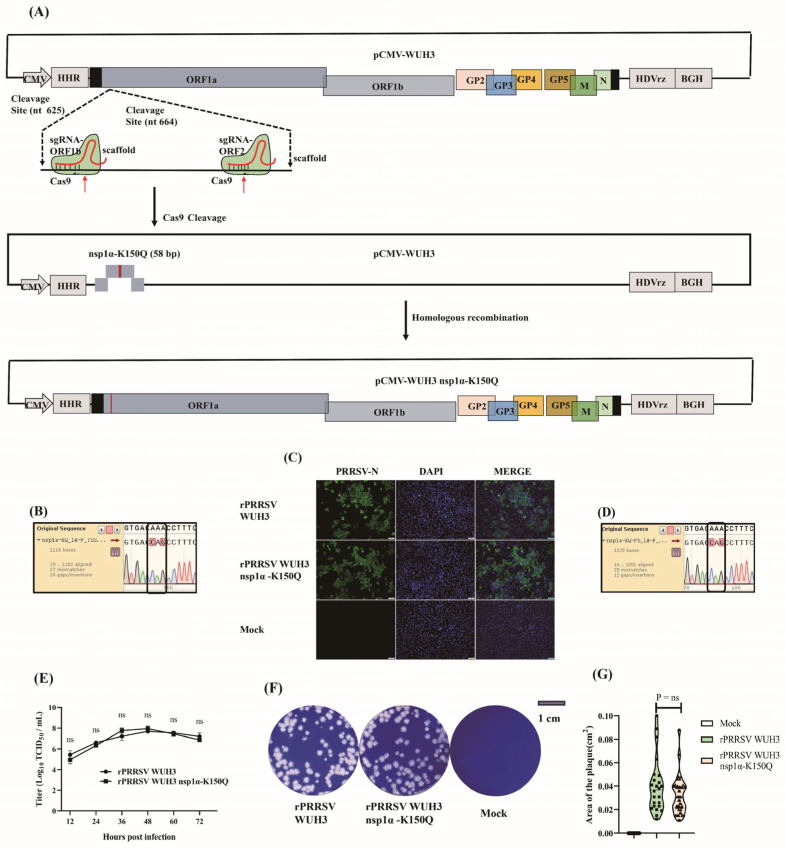
Site-specific mutation of PRRSV genome using CRISPR/Cas9. (**A**) Schematic diagram of mutating lysine at the position of 150 in nsp1α of PRRSV WUH3 using CRISPR/Cas9. (**B**) Confirmation of the constructed pCMV-WUH3 nsp1α-K150Q using Sanger sequencing. (**C**) The detection of rPRRSV WUH3 nsp1α-K150Q using IFA. MARC-145 cells were transfected with pCMV-WUH3 nsp1α-K150Q and pCMV-WUH3 for 5 days, followed by IFA to detect the expression of PRRSV-N protein. (**D**) Confirmation of the K150Q mutation in nsp1α of rPRRSV using Sanger sequencing. (**E**) The multiple-step growth curves of rPRRSV WUH3 nsp1α-K150Q and rPRRSV WUH3 in MARC-145 cells at an MOI of 0.1 were analyzed using TCID_50_ assays. (**F**) Plaques formed by rPRRSV WUH3 nsp1α-K150Q and rPRRSV WUH3 in MARC-145 were stained with 1% crystal violet at 96 hpi. (**G**) Areas of plaques formed by rPRRSV WUH3 nsp1α-K150Q and rPRRSV WUH3 were detected using Image J and compared using GraphPad Prism 7, respectively. Data are displayed in a Violin Plot. ns, *p* > 0.5.

We followed a two-step process to introduce the K150Q mutation in PRRSV nsp1α. Initially, we synthesized two sgRNAs targeting nsp1α using ssDNA-scaffold, ssDNAa-nsp1α, and ssDNAb-nsp1α as listed in Table 2. These two sgRNAs only possess a short separation spacer of 20 bp. Guided by these sgRNAs, Cas9 cleaved the pCMV-WUH3 at two adjacent positions around K150 of the nsp1α to linearize pCMV-WUH3. Next, a short and double-stranded oligonucleotide fragment containing the desired K150Q mutation and the linearized pCMV-WUH3 were ligated using homologous recombination to form pCMV-WUH3 nsp1α-K150Q, which was subsequently confirmed using Sanger sequencing (Figure 6A,B). The pCMV-WUH3 nsp1α-K150Q was transfected into MARC-145 cells to generate rPRRSV WUH3-nsp1α-K150Q; then, the typical PRRSV CPE was observed at approximately 5 days post-transfection. Additionally, the expression of PRRSV-N protein in cells transfected with pCMV-WUH3 nsp1α-K150Q was confirmed by IFA (Figure 6C). We also performed Sanger sequencing to confirm the mutation K150Q in the nsp1α of rPRRSV WUH3 nsp1α-K150Q (Figure 6D). The multiple-step growth curves showed that rPRRSV WUH3 nsp1α-K150Q and rPRRSV WUH3 possessed similar growth kinetics, and both reached the peak of proliferation at 48 hpi (Figure 6E). Moreover, the results of plaque assays demonstrated that no significant difference existed in the size of plaques formed by rPRRSV WUH3 nsp1α-K150Q and rPRRSV WUH3 (Figure 6F,G). Collectively, we developed an efficient technology for the site-directed mutation of the PRRSV genome.

## 4. Discussion

PRRSV has been devasting the swine industry for more than thirty years due to its high antigenic variability and immunosuppressive properties [42,43]. Therefore, it is crucial to comprehensively understand viral pathogenesis and develop broad-spectrum vaccines. Herein, we developed an efficient reverse genetics system for PRRSV and demonstrated its potential application in vaccine development and viral gene function study [9,44,45,46].

Traditionally, the full-length infectious cDNA clone of PRRSV is constructed by gradually ligating viral genome fragments into a vector, which is time-consuming and laborious [47]. To avoid these problems, we analyzed the distribution of restriction enzymes in the genome of PRRSV WUH3. Finally, we screened an applicable restriction enzyme, BstXI, which can digest the viral genome into six gene fragments. These six gene fragments were then assembled into a full-length infectious cDNA clone using a one-step-assembly method. Compared to the previously reported methods, this approach is time-saving and convenient.

Previously reported methods for RNA virus genome manipulation usually face the challenge of selecting suitable restrictive endonucleases [23,47]. In contrast, the CRISPR/Cas9-based method improves the flexibility of editing the genome and saves time when modifying the viral genome [37,38]. In this study, this method was used to insert the exogenous gene (EGFP) or induce a site-directed mutation (nsp1α-K150Q) in the genome of PRRSV WUH3, thus, successfully generating rPRRSV WUH3-EGFP and rPRRSV WUH3 nsp1α-K150Q, respectively. Altogether, we developed a strategy for editing the PRRSV genome in vitro using CRISPR/Cas9 technology.

The region in the viral genome where the exogenous gene is inserted is key to the stability of recombinant viruses. A previous study revealed that the recombinant PRRSV carrying a foreign HA tag fused to ORF7 loses the introduced epitope as early as the second passage [48]. Similarly, when the GFP gene is fused to the nsp2 gene of PRRSV, the green fluorescence is no longer observed after seven passages [25]. Additionally, a rescued PRRSV carrying a GFP gene at the interval between ORF4 and ORF5 only maintains the expression of GFP within eight passages [26]. However, the exogenous gene placed into the interval between ORF1b and ORF2 of PRRSV continues to express exogenous protein for 37 passages [49]. So, we used CRISPR/Cas9 to insert an EGFP gene into the interval between ORF1b and ORF2 of PRRSV WUH3 and acquired an EGFP-labeled PPRSV (rPRRSV WUH3-EGFP).

The construction of site-mutated viruses involves two steps, namely, which amino acid can be mutated and how to construct the recombinant virus. The target residue was always mutated into alanine previously, but random mutation in amino acid may be lethal to a recombinant virus [50]. Thus, we aligned the gene sequences of nsp1α in different PRRSV strains and found that the residue at position 150 in nsp1α is mostly K in NA-type PRRSV and Q in EU-type PRRSV. Based on this result, we determined to mutate K150 in PRRSV WUH3 nsp1α into Q150. Moreover, two sgRNAs separated by only 20 bp were used to guide Cas9; then, we found that Cas9 could still efficiently cleave pCMV-WUH3. This suggests that the in vitro cleavage ability of Cas9 seems not to be affected by the distance between sgRNAs, which is consistent with the in vivo results reported previously [51]. Significantly, the shorter the distance between sgRNAs, the shorter the oligonucleotide fragment used for substitutions, which can remarkably reduce the synthesis cost of the oligonucleotide fragment and be more time-saving. Finally, the rPRRSV WUH3 nsp1α-K150Q virus was successfully acquired, which may be a crucial tool for subsequent studies on the function of K150 in PRRSV nsp1α.

In summary, a convenient PRRSV-genome-manipulating method based on CRISPR/Cas9 was established in this study. It could be used for inserting foreign genes into viral genomes and mutating viral genes precisely as well. This convenient method will certainly boost the development of enhanced vaccines in the future and accelerate the study on viral pathogenesis.

## Figures and Tables

**Table 1:** The primers used for assembling pCMV-WUH3 in this study.

Primer Name	Sequence (5′–3′)
WUH3-F1	ATGACGTATAGGTGTTGGCTCTA
WUH3-R1	GCAACGTCCACCGGAGTGGCTC
WUH3-F2	GAGCCACTCCGGTGGACGTTGC
WUH3-R2	ACCATCCGGTTCGCGATGGCG
WUH3-F3	CGCCATCGCGAACCGGATGGT
WUH3-R3	CTTTAGTCCATTCAGCTGGGC
WUH3-F4	GCCCAGCTGAATGGACTAAAG
WUH3-R4	CTCCAGTTCTTTGGCAGTC
WUH3-F5	GACTGCCAAAGAACTGGAG
WUH3-R5	CACAGCAAGATAGAACGGCAC
WUH3-F6	TGTGTGCGTCAACTTTACC
WUH3-R6	TTTTTTTTTTAATTACGGCCGCATGGTTCTC
pCMV-F	CATGCGGCCGTAATTAAAAAAAAAAAAAAAAAAAAAAAAAAAGGCCGGCATGG
pCMV-R	TAGAGCCAACACCTATACGTCATCCGACGGTACCGGGTACCGTTTC

**Table 2:** The oligos used for constructing rPRRSV WUH3-EGFP and rPRRSV WUH3 nsp1α-K150Q in this study.

Oligo Name	Sequence (5′–3′)
ssDNAa-ORF1b	TTAATACGACTCACTATAGGGATGTCAAAGGTACCACCGTGTTTTAGAGCTAGA
ssDNAb-ORF2a	TTAATACGACTCACTATAGGGAAGAGTACAAGAAGCTGCAAGTTTTAGAGCTAGA
ORF1b-F	CATCGGCGATGTCAAAGGTACC
ORF1b-mR	CCTCGCCCTTGCTCACCATTCAATTCAGGCCTAAAGTTG
EGFP-F	CAACTTTAGGCCTGAATTGAATGGTGAGCAAGGGCGAGG
EGFP-R	CGTTCCGCTGAAACTCTGGTTAAAGGGGTTGCCGCGGAACTTACTTGTACAGCTCGTCCATGC
ORF2-mF	CCAGAGTTTCAGCGGAACGAATGAAATGGGGTCTATGCAAAGC
ORF2-R	GCACAACAAAAAGAGTACAAGAAGCTGC
ssDNA-scaffold	AAAAGCACCGACTCGGTGCCACTTTTTCAAGTTGATAACGGACTAGCCTTATTTTAACTTGCTATTTCTAGCTCTAAAAC
ssDNAa-nsp1α	TTAATACGACTCACTATAGGGGTTTGTCACTCACATGCAGTTTTAGAGCTAGA
ssDNAb-nsp1α	TTAATACGACTCACTATAGGGTAACACATGAGTTGCTCCCGTTTTAGAGCTAGA
nsp1α-K150Q-F	CGCCAACTCCCTGCATGTGAGTGACCAGCCTTTCCCGGGAGCAACTCATG
nsp1α-K150Q-R	GGTTAACACATGAGTTGCTCCCGGGAAAG

## Data Availability

The datasets generated in this study are available upon request from the corresponding authors.
